# STON2 negatively modulates stem-like properties in ovarian cancer cells via DNMT1/MUC1 pathway

**DOI:** 10.1186/s13046-018-0977-y

**Published:** 2018-12-05

**Authors:** Shanshan Xu, Yongfang Yue, Songfa Zhang, Caiyun Zhou, Xiaodong Cheng, Xing Xie, Xinyu Wang, Weiguo Lu

**Affiliations:** 1 Department of Gynecologic Oncology; Women’s Hospital, School of Medicine, Zhejiang University, Hangzhou, 310006 China; 2 Department of Pathology, Women’s Hospital, School of Medicine, Zhejiang University, Hangzhou, 310006 China; 3 Women’s Reproductive Health Laboratory of Zhejiang Province; Women’s Hospital; School of Medicine, Zhejiang University, Hangzhou, 310006 China

**Keywords:** Ovarian cancer, Cancer stem cell, STON2, MUC1, DNMT1

## Abstract

**Background:**

Cancer stem cells (CSCs) possess abilities of self-renewal and differentiation, have oncogenic potential and are regarded to be the source of cancer recurrence. However, the mechanism by which CSCs maintain their stemness remains largely unclear.

**Methods:**

In this study, the cell line-derived ovarian CSCs (OCSCs), 3AO and Caov3, were enriched in serum-free medium (SFM). Differentially expressed proteins were compared between the OCSC subpopulation and parental cells using liquid chromatography (LC)-mass spectrometry (MS)/MS label-free quantitative proteomics. Sphere-forming ability assays, flow cytometry, quantitative real-time polymerase chain reaction (qPCR**)**, western blotting, and in vivo xenograft experiments were performed to evaluate stemness. RNA-sequencing (RNA-seq) and pyrosequencing were used to reveal the mechanism by which STON2 negatively modulates the stem-like properties of ovarian cancer cells.

**Results:**

Among the 74 most differentially expressed proteins, stonin 2 (STON2) was confirmed to be down-regulated in the OCSC subpopulation. We show that STON2 negatively modulates the stem-like properties of ovarian cancer cells, which are characterized by sphere formation, a CD44^+^CD24^−^ ratio, and by CSC- and epithelial mesenchymal transition (EMT)-related markers. *STON2* knockdown also accelerated tumorigenesis in NOD/SCID mice. Further investigation revealed a downstream target, mucin 1 (MUC1), as up-regulated upon the down regulation of STON2. A decrease in both DNA methyltransferase 1 (DNMT1) expression and methylation in the promoter region of *MUC1* was associated with subsequently elevated *MUC1* expression, as detected in *STON2* knockdown in 3AO and Caov3 cells. Direct *DNMT1* knockdown simultaneously elevated *MUC1* expression. The functional significance of this STON2-DNMT1/MUC1 pathway is supported by the observation that *STON2* overexpression suppresses MUC1-induced sphere formation of OCSCs. The paired expression of STON2 and MUC1 in ovarian cancer specimens was also detected revealing the prognostic value of STON2 expression to be highly dependent on MUC1 expression.

**Conclusions:**

Our results imply that STON2 may negatively regulate stemness in ovarian cancer cells via DNMT1-MUC1 mediated epigenetic modification. STON2 is therefore involved in OCSC biology and may represent a therapeutic target for innovative treatments aimed at ovarian cancer eradication.

**Electronic supplementary material:**

The online version of this article (10.1186/s13046-018-0977-y) contains supplementary material, which is available to authorized users.

## Background

Ovarian cancer represents the most lethal of all gynecological neoplasms. The American Cancer Society predicts that 22,440 women will be diagnosed with ovarian cancer and 14,070 women will die from the disease in 2018. Cancer statistics gathered over the period from 2006 to 2012 suggest that the overall 5-year survival rate of ovarian cancer patients is 47%. However, in advanced stage patients this value drops to 29% [1]. Although 70% patients with ovarian cancer respond to first-line chemotherapy, most of them ultimately suffer from recurrence and metastasis [2]. It is noteworthy that these survival statistics have hardly changed over time, despite the introduction of platinum-based treatment more than 30 years ago [3]. Emerging evidence has confirmed that a small population of tumor cells with stemness properties, now known as CSCs or cancer stem-like cells (CSLCs), occur in cancer tissues [4]. These cells possess abilities of self-renewal and differentiation, have strong oncogenic potential and are considered as the source of cancer recurrence [5]. Thus, CSC-targeted therapy is expected to considerably improve the prognosis of patients with ovarian cancer [6]. Unfortunately, identification and eradication of CSCs has proved to be far from optimal [7]. Persistent exploration of CSC characteristics may be beneficial for the development of novel CSC-targeting drugs.

Since these cells were first isolated, cloned, and propagated in vitro*,* OCSCs have been widely recognized by their stemness or progenitor-like properties, which include sphere formation, self-renewal and tumorigenic abilities [8]. Previously, we have successfully enriched the OCSC subpopulation using a sphere formation model and observed that CD44^+^CD24^−^ cells are highly tumorigenic [9]. Furthermore, we verified that cyclin D1 affects EMT in OCSLCs [10], whereas in other studies, NANOG and c-MYC were reported to be involved in OCSC regulation and acted as cancer stem related-markers [11, 12]. To gain deeper insight into the molecular basis for OCSCs, we used LC-MS/MS label-free quantitative proteomics and bioinformatic analysis to identify the key factors that are differentially down-regulated in the OCSC subpopulation. STON2, an endocytic sorting adaptor [13], was of particular interest. *STON2* knockdown promoted OCSC stemness.

Recent analysis in the Kyoto Encyclopedia of Genes and Genomes (KEGG) revealed that OCSC-specific gene expressions are enriched in the endocytosis pathway [14]. Many of these genes were noted to be involved in key steps of endocytosis related to the resurrection, multidrug resistance, stemness maintenance of CSCs [15, 16]. Here, we present novel observations, which indicate that STON2 is involved in modulating stemness in ovarian cancer cells.

The oncogenic MUC1, a member of the class of epigenetically controlled genes, is a transmembrane protein that is aberrantly overexpressed and confers poor prognosis in a variety of cancers, including pancreatic, colorectal, breast, lung and ovarian cancer [17]. Increasing evidence has shown that MUC1 is also associated with the stemness of lung cancer [18] and breast cancer [19, 20]. High expression levels of MUC1 are well documented as correlated with metastasis, chemoresistance, and the survival of ovarian cancer cells [21, 22]. However, the regulatory mechanisms of MUC1 in ovarian cancer remain elusive.

In this study, using RNA-seq and gene function experiments, we identify that MUC1 acts as a downstream target for STON2, and modulates stem-like properties. Interestingly, MUC1 levels are elevated by CpG demethylation in cancer cells, where promoter methylation plays an important role in determining *MUC1* expression [23, 24]. We provide evidence that STON2-regulated *MUC1* expression might be mediated by DNMT1-induced methylation in the promoter region of *MUC1*. The higher expression of STON2 together with lower expression of MUC1 is associated with a favorable prognosis. STON2, therefore, is involved in OCSC biology and may represent a therapeutic target for innovative treatments aimed at ovarian cancer eradication.

## Methods

### Cell culture and sphere-forming ability assay

The human epithelial ovarian cancer cell line, Caov3, was obtained from the American Type Culture Collection (ATCC, Manassas, Virginia, USA). The human ovarian adenocarcinoma cell line 3AO was acquired from the Women’s Hospital, School of Medicine, Zhejiang University, where it was tested and authenticated. It was not cultured continuously for more than 3 months. Adherent Caov3 cells were cultured in their standard medium as recommended by ATCC. 3AO cells were cultured in Roswell Park Memorial Institute (RPMI)-1640 medium (BI, Kibbutz Beit-Haemek, Israel), supplemented with 10% fetal bovine serum (FBS) (Invitrogen, New York, USA), maintained at 37 °C in 5% CO_2_ and detached using trypsin/EDTA solution. The anchorage-independent spheroids were generated from Caov3 or 3AO cells after plating, 5 × 10^4^ cells per well, in ultra-low attachment 6-well culture plates (Corning, New York, USA) and culturing in a SFM composed of Dulbecco’s modified Eagle’s medium (DMEM)/F12 (BI, Kibbutz Beit-Haemek, Israel), 10 ng/mL basic fibroblast growth factor, 20 ng/mL epidermal growth factor (PeproTech, Rocky Hill, USA), 1 mg/mL insulin (Sigma-Aldrich, Burlington, MA, USA), 10 μL/mL B27 additive (Life Technologies, Carlsbad, California, USA), 100 U/mL penicillin, and 100 μg/mL streptomycin. Fresh medium was added after every two or three days and the cultures were maintained at 37 °C in 5% CO_2_ for 7 days. During the passaging of spheroids, the cells were centrifuged and digested with trypsin, and cultured in SFM.

After plating 400 cells per well in ultra-low attachment 96-well culture plates (Corning, New York, USA), Caov3 or 3AO cells were cultured in a SFM composed of DMEM/F12 (BI, Kibbutz Beit-Haemek, Israel), 10 ng/mL basic fibroblast growth factor, 20 ng/mL epidermal growth factor (PeproTech, Rocky Hill, USA), 1 mg/mL insulin (Sigma-Aldrich, Burlington, MA, USA), 10 μL/mL B27 additive (Life Technologies, Carlsbad, California, USA), 100 U/mL penicillin, and 100 μg/mL streptomycin. Fresh medium was added after every two or three days and the cultures were maintained at 37 °C in 5% CO_2_ for 7 days.

### LC-MS/MS label-free quantitative proteomics and bioinformatic analysis

Parental and spheroids of 3AO cells (three independent samples in each group) were cracked in 200 μl lysis buffer, disrupted with agitation, boiled for 10 min, and centrifuged at 12000 rpm for 10 min. The supernatant was then collected and digested overnight at 37 °C with trypsin (Promega, Madison, Wisconsin, USA). The peptide of each sample was desalted on C18 cartridges (Sigma, Burlington, MA, USA) and then resuspended in 0.1% trifluoroacetic acid after centrifugation. MS analysis was performed on a Finnigan LTQ VELOS MS (ThermoFisher, Waltham, MA, USA). Each scan cycle included one full MS1 scan in profile mode and 20 MS2 scans in centroid mode. The peptides were investigated automatically according to the MS/MS spectra in the International Protein Index (IPI) human protein database using the Bioworks Browser software suite (Thermo, Waltham, MA, USA). The quantification of peptides was performed using the DeCyder differential analysis software (GE Healthcare, Munich, Germany).

### Plasmids, siRNA transfection, and lentivirus infection

FLAG-tagged full length open reading frames (ORFs) of human STON2 (NM_033104.3) were ligated into a pcDNA3.1 vector (BioVector, Beijing, China) after HindIII and XhoI digestion. HA-tagged human MUC1 (NM_001204286.1) was cloned into a pcDNA3.1 vector (BioVector, Beijing, China) after HindIII and EcoRI digestion. The sequences of all the constructs were verified by DNA sequencing. The primer sequences are listed in Additional file 1: Table S1. Transfections were performed using X-tremeGENE HP DNA transfection reagent (Roche, Basel, Switzerland) following the manufacturer’s protocols.

The short-hairpin RNAs (shRNAs) for STON2 and MUC1 oligonucleotides were cloned into hU6-MCS-CMV-puromycin lentivirus expression vectors between the AgeI and EcoRI sites and then transfected into adherent cells according to instructions. After 72 h of transfection, the cells were selected using 5 μg/ml puromycin for 4 days. All construct sequences were confirmed using DNA sequencing. The shRNA oligonucleotides are listed in Additional file 2: Table S2.

Small-interfering RNAs (siRNAs) against STON2 and DNMT1*,* along with RNAi negative controls, were purchased from Genepharma (Shanghai, China). Cells were transfected with the siRNAs (50 nM) using Lipofectamine™ RNAiMAX (ThermoFisher, Waltham, MA, USA) following the manufacturer’s protocols. The siRNA sequences are listed in Additional file 2: Table S2.

### RNA extraction and qPCR

Total RNA was extracted using an RNA extraction kit (TaKaRa, Dalian, China) and reverse-transcribed into cDNA using the reverse transcription cDNA kit (TAKAR, Dalian, China). PCR reactions were performed using SYBR® Premix Ex Taq™ (TaKaRa, Dalian, China) and applied biosystems 7900HT fast real-time PCR system (Life Technologies, Carlsbad, California, USA). The relative mRNA expression was calculated using the 2^-∆∆Ct^ method and normalized to *GAPDH* expression. Primer sequences are shown in Additional file 1: Table S1.

### Western blot analysis

Cells were lysed using a radioimmunoprecipitation assay (RIPA) lysis buffer (Beyotime, Shanghai, China) supplemented with PMSF inhibitor (Beyotime, Shanghai, China). Protein lysates were loaded and separated on a 10% sodium dodecyl sulfate polyacrylamide gel and transferred onto 0.22-μm polyvinylidene fluoride (PVDF) membranes (Bio-Rad, Hercules, California, USA). The membranes were then blocked with Tris buffered saline Tween 20 (TBST) containing 5% non-fat milk for 1 h, and probed with primary antibodies overnight at 4 °C. They were then washed thrice with TBST for 10 min each and probed with secondary antibodies for 1 h, followed by washing three times in TBST for 10 min per wash. The bands were visualized using an enhanced chemiluminescence (ECL) kit (ThermoFisher, Waltham, MA, USA) in Image quant LAS400 mini (GE Healthcare, Munich, Germany). Primary antibodies against STON2 (Santa, Dallas, Texas), MUC1 (ThermoFisher, Waltham, MA, USA), NANOG (Sigma, Burlington, MA, USA), c-MYC (CST, Danvers, MA, USA), DNMT1 (Abcam, Cambridge, MA, USA), DNMT3A (Abcam, Cambridge, MA, USA), DNMT3B (Abcam, Cambridge, MA, USA), E-cadherin (Abcam, Cambridge, MA, USA), N-cadherin (CST, Danvers, MA, USA), and fibronectin (Abcam, Cambridge, MA, USA) were used. GAPDH (Abcam, Cambridge, MA, USA) was the loading control.

### Flow cytometry analysis

Spheroids were centrifuged and digested with trypsin, after which 1 × 10^5^ cells were counted and resuspended in 100 μl PBS, stained with either anti-CD44-FITC (eBiosciences, Vienna, Austria), anti-CD24-APC (eBiosciences, Vienna, Austria) antibodies or isotype control antibodies (eBiosciences, Vienna, Austria), and incubated for 30 min at room temperature following the manufacturer’s protocol. They were then detected using a FC 500 series flow cytometer (Beckman Coulter, Brea, CA, USA), and the data was subsequently analyzed using the CXP 2.1 software.

### RNA-seq analysis

3AO cells transfected with shSTON2 lentivirus or control shRNA were cultured in a SFM for 7 days. Total RNA was prepared from spheroids using an RNeasy mini kit (Qiagen, Dusseldorf, Germany) according to the manufacturer’s instructions. RNA quality was evaluated using a RNA Nano 6000 assay kit of the Bioanalyzer 2100 system (Agilent Technologies, CA, USA). High-quality RNA from shSTON2 and control groups was used for transcriptome sequencing (three independent samples for each group). The libraries were sequenced on an Illumina HiSeq platform using a 125 bp/150 bp paired-end sequencing strategy. The original image data generated by the sequencing machine was converted into sequence data via base calling using a TruSeq PE cluster kit v3-cBot-HS (Illumia, San Diego, CA, USA). These were then subjected to standard quality controls. Clean data was obtained by removing reads containing adapter, poly-N, and low quality reads from the raw data. Next, the Q20, Q30, and GC contents of the clean data were calculated. Paired-end clean reads were aligned to the reference genome using STAR. Read numbers mapped to each gene were counted by HTSeq v0.6.0. Differential expression analysis of two conditions was performed using the DESeq2 R package (1.10.1). The resulting *P*-values were adjusted using the benjamini and hochberg’s approach for controlling the false discovery rate (FDR). Genes with an adjusted *P*-value< 0.05, as identified by DESeq2, were considered to be differentially expressed.

### DNA isolation, sodium bisulfite conversion, and pyrosequencing

Genomic DNA was extracted using a QIAamp DNA mini kit (Qiagen, Dusseldorf, Germany). One microgram of genomic DNA was bisulfite-modified using an EpiTect bisulfite kit (Qiagen, Dusseldorf, Germany) following the manufacturer’s protocol. The modified DNA was used as a template for subsequent PCR. Primers for PCR amplification and sequencing were designed using the pyromark assay design 2.0 software (Qiagen, Dusseldorf, Germany). A Hotstar Taq DNA polymerase PCR kit (Qiagen, Dusseldorf, Germany) was used for the experiments under the following conditions: 95 °C 3 min; 40 cycles of 94 °C 30 s, 52 °C 30 s, 72 °C for 1 min; 72 °C for 7 min. The acquisitions were assessed using gel electrophoresis and pyrosequenced with pyromark Q96 ID (Qiagen, Dusseldorf, Germany). The primers for pyrosequencing are shown in Additional file 1: Table S1.

### Patient specimen selection and immunohistochemistry

With the permission of the patients and the ethical committee of the Women’s Hospital, School of Medicine, Zhejiang University, 165 formalin-fixed and paraffin-embedded tissue samples were obtained from patients that had been diagnosed with ovarian serous, mucinous, endometrioid, clear-cell and mixed carcinomas from January 2002 to December 2009. These were used for immunohistochemical analyses. All patients underwent primary surgery, followed by paclitaxel-based chemotherapy. The deadline of the follow-up was July 30, 2017. As 20 patients failed to re-contact, only the remaining 145 patients were used for the calculation of progression-free survival (PFS) and overall survival (OS). All pathological diagnosis was reconfirmed blindly by an expert pathologist. The section slides were counter-stained with haematoxylin, dehydrated and mounted. The details were described as previously [25]. Anti-STON2 (Abcam, Cambridge, MA, USA) (1:200) and anti-MUC1 (Abcam, Cambridge, MA, USA) (1:500) were used for IHC. Five microscope fields were picked for evaluating the semiquantification of STON2 and MUC1 staining. The intensity of immunostaining was graded as follows: 1+, weak; 2+, moderate; 3+, strong or 4+, very strong (Additional file 3: Figure S1). The area of positive cancer cells in was categorized as follows: 1+, 0 to 25%; 2+, 25 to 50%; 3+, 50 to 75% or 4+, 75 to 100%. The score for each microscopic field was obtained by multiplying the two parts of score, The sum was acquired by adding the scores of the five microscopic fields. The sum from 0 to 42 was assigned as “low expression”, from 43 to 80 was assigned as “high expression” [26].

### In vivo xenograft experiments

All animal experiments were performed in accordance with Animal Research Reporting In Vivo Experiments (ARRIVE) guidelines for the use of laboratory animals and were approved by the ethics committee of Zhejiang University. NOD/SCID mice (female, 4–6-week-old) were purchased from the Chinese Academy of Medical Sciences (Beijing, China) and randomly assigned to each treatment group. For experiments, 1 × 10^5^ or 1 × 10^4^ 3AO spheroids transfected with shSTON2 or shNC were resuspended in 100 μl PBS and injected into the right flank of mice, which were then monitored weekly for 4 weeks. The tumor volumes were calculated according to the formula V = a × b^2^ × 0.5, where a is the largest diameter and b is the smallest diameter of the tumor.

### Statistical analysis

Differences in clinicopathological characteristics were assessed using the chi-squared test. Cumulative survival probabilities were calculated using the Kaplan-Meier method. Survival rates were compared using the log-rank test. Two-tailed Student’s t-tests were used to perform a statistical comparison between two groups unless otherwise indicated. Statistical tests were performed using the SPSS software, version 20.0 (SPSS Inc.) or with GraphPad Prism 6.0 (GraphPad Software, Inc.). The level of statistical significance was set at *P < 0.05, **P < 0.01, ***P < 0.001.

## Results

### STON2 expression is down-regulated in an OCSC-enriched population

**Fig. 1:**
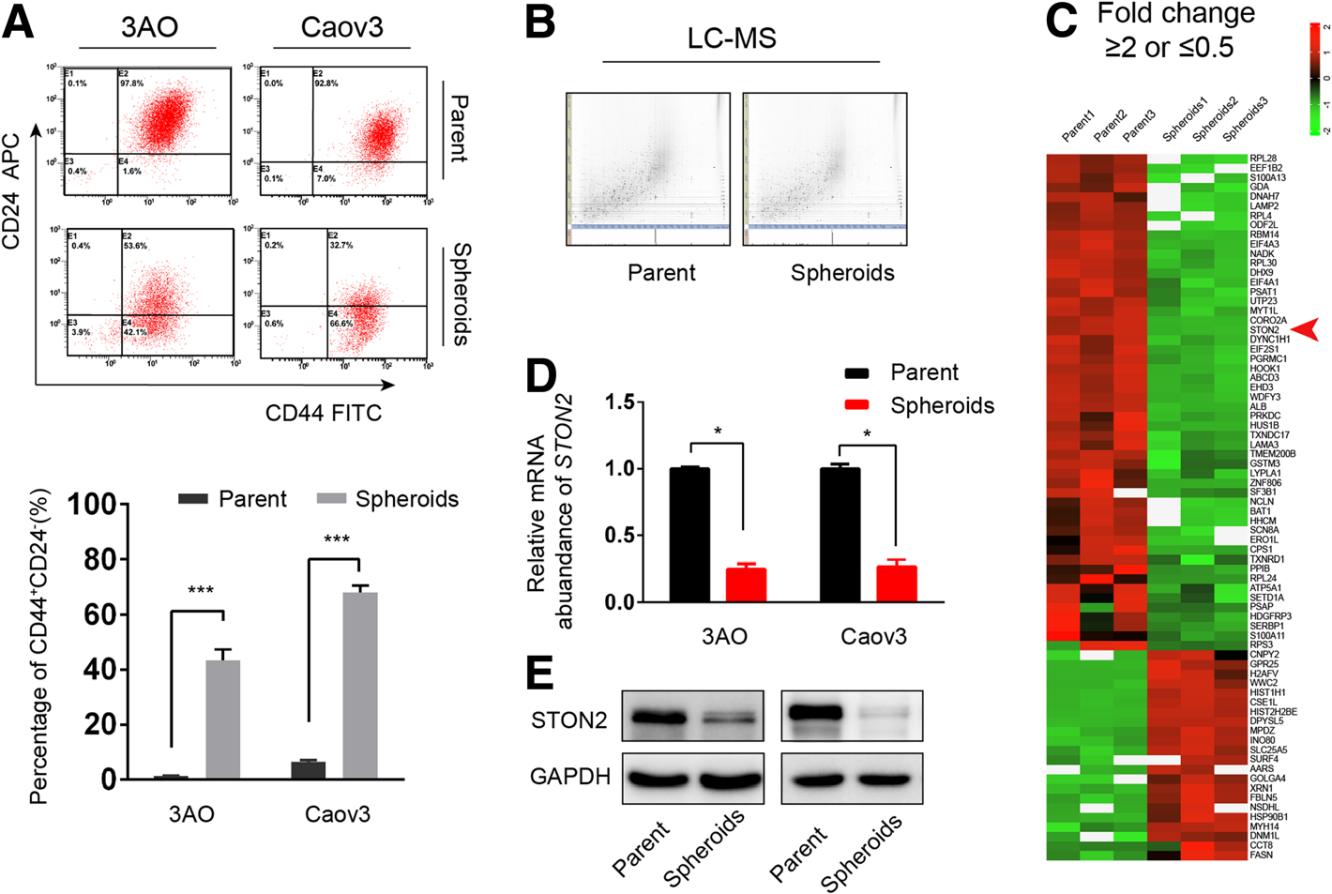
STON2 expression is down-regulated in an OCSC-enriched population**. a** 3AO and Caov3 cells were cultured in spheroid culture conditions to enrich OCSCs. The CD44^+^CD24^−^ cell population were analyzed by FCM. The parental cells in normal culture were used as the control. **b** 2D feature maps of LC-MS/MS data from the analysis between parental cells and spheroids of 3AO cells. The horizontal axis shows retention time, the vertical axis shows mass-to-charge ratio, and the gray color level indicates the intensity value. **c** Protein cluster map generated by Cluster software (FDR < 0.05, fold change ≥2 or ≤ 0.5). Proteins up-regulated in spheroids are shown in red, and the down-regulated proteins in green. The intensity of the color green or red corresponds to the degree of alteration, according to the color strip at the right of the figure. The arrow indicates STON2. **d** qPCR analysis of *STON2* expression in parental cells and spheroids. **e** Immunoblot analysis of STON2 expression in parental cells and spheroids. Data represents the mean ± S.E. of three independent experiments. The level of significance is indicated by *P < 0.05, ***P < 0.001

The ovarian cancer cells were cultured in suspension culture conditions to enrich the spheroids [9]. As shown in Fig. 1a, spheroids with considerably increased CD44^+^CD24^−^ subpopulation are characterized as CSCs and referred to as OCSCs for the subsequent experiments. Using proteomics, we analyzed the differential protein expression between parental cells and spheroids from 3AO cells. The 2-dimensional image generated by LC-MS/MS analysis (Fig. 1b) revealed that 685 peptides and 187 nonredundant peptides had been differentially expressed between parental cells and spheroids (FDR < 0.05, Additional file 4: Table S3). The heat map of Fig. 1c shows 74 of the most differentially expressed proteins in DeCyder differential analysis (FDR < 0.05, fold change ≥2 or ≤ 0.5). Notably, qPCR revealed a lesser STON2 expression in the spheroids than in the parental cells (Fig. 1d). Western blotting further validated the significantly lower expression of STON2 protein in the spheroids than in parental 3AO and Caov3 cells (Fig. 1e).

### STON2 negatively modulates stem-like properties in ovarian cancer cells

**Fig. 2:**
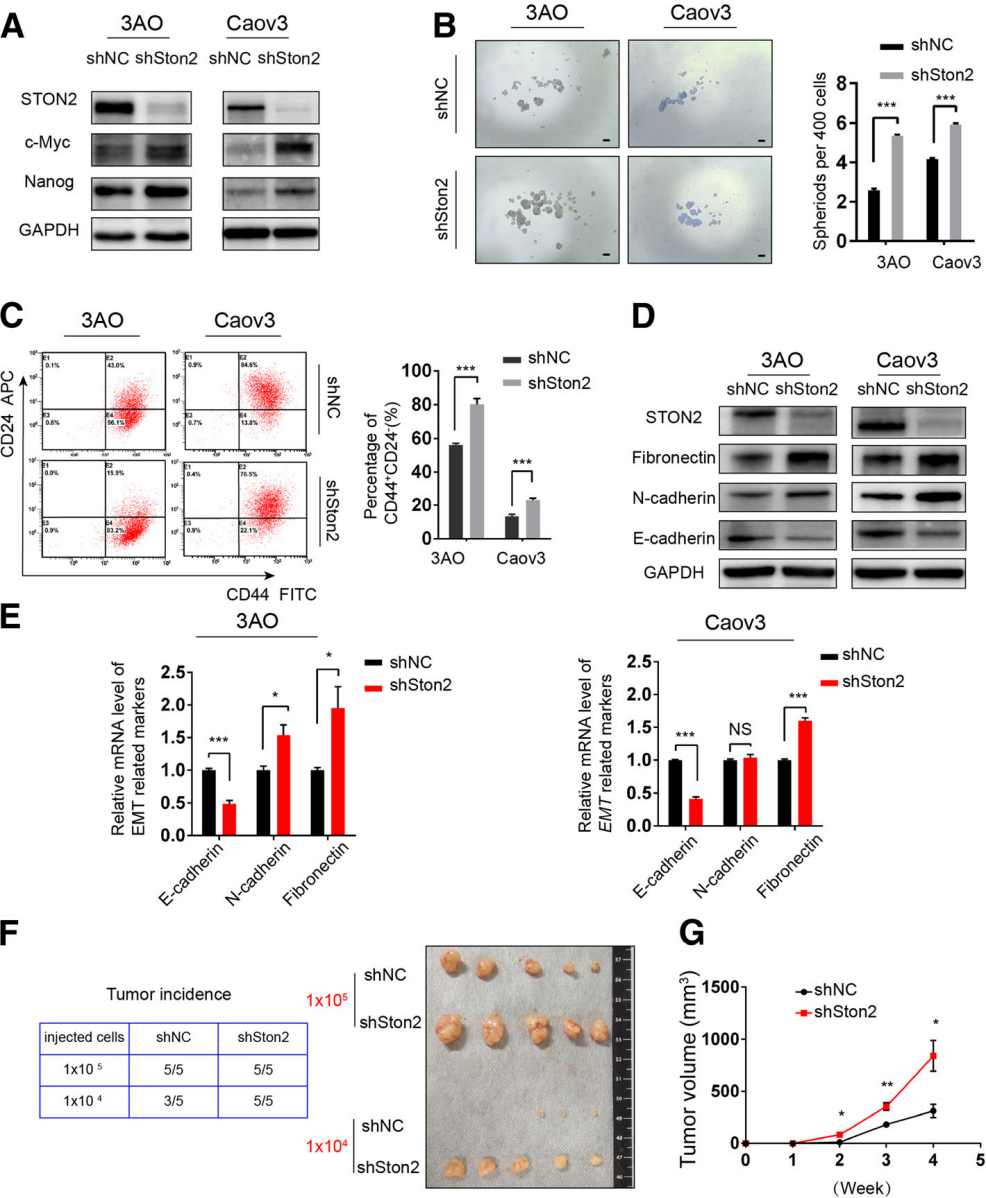
STON2 knockdown promotes stem-like properties of ovarian cancer cells. **a** 3AO and Caov3 cells were transfected with a STON2-specific shRNA for 48 h, and cultured in spheroid culture conditions for 7 days. The expressions of STON2 and the CSC-related markers NANOG and c-MYC were detected by immunoblot analysis. **b** Quantitation and representative images of sphere-formation (sphere > 50 μm), Scale bars, 50 μm. **c **The effect of STON2 knockdown on the CD44^+^CD24^−^ phenotype was analyzed using FCM. **d** Immunoblot analysis of EMT-related markers (E-cadherin, N-cadherin, and fibronectin). **e** qPCR analysis of EMT-related markers (E-cadherin, N-cadherin, and fibronectin). **f**, **g** 3AO cells were transfected with shNC or shSTON2 for 48 h, cultured in spheroid culture conditions for 7 days, and then injected into NOD/SCID mice (*n* = 5 in each group). All mice were sacrificed at week 4 and the tumor incidence evaluated (left panel). Subcutaneous tumors are shown (right panel) (**f**).Tumor diameters of xenografts from mice injected with 1 × 10^5^ cells were measured at a regular intervals of 1 week for up to 4 weeks and the tumor volume was calculated (**g**). Data represents mean ± S.E. of three independent experiments. The level of significance is indicated by *P < 0.05, **P < 0.01, ***P < 0.001

To explore the function of STON2 in ovarian cancer, a shRNA was used to suppress STON2 expression in 3AO and Caov3 cells cultured in SFM for 7 days. STON2 protein levels were successfully inhibited by specific shRNA (Fig. 2a). We observed that STON2 knockdown up-regulated the expression of stem cell related-markers such as NANOG and c-MYC [27] (Fig. 2a) and that spheroid numbers were significantly higher in OCSCs than in shNC cells (Fig. 2b). Flow cytometry analysis showed that the proportion of CD44^+^CD24^−^ cells had also increased (Fig. 2c). We quantified both the protein and mRNA levels of EMT-related key factors, including E-cadherin, N-cadherin, and fibronectin, all of which revealed significant differences in expressions between shSTON2 and shNC cells, except the N-cadherin mRNA expression in Caov3 line (Fig. 2d, e).

**Fig. 3:**
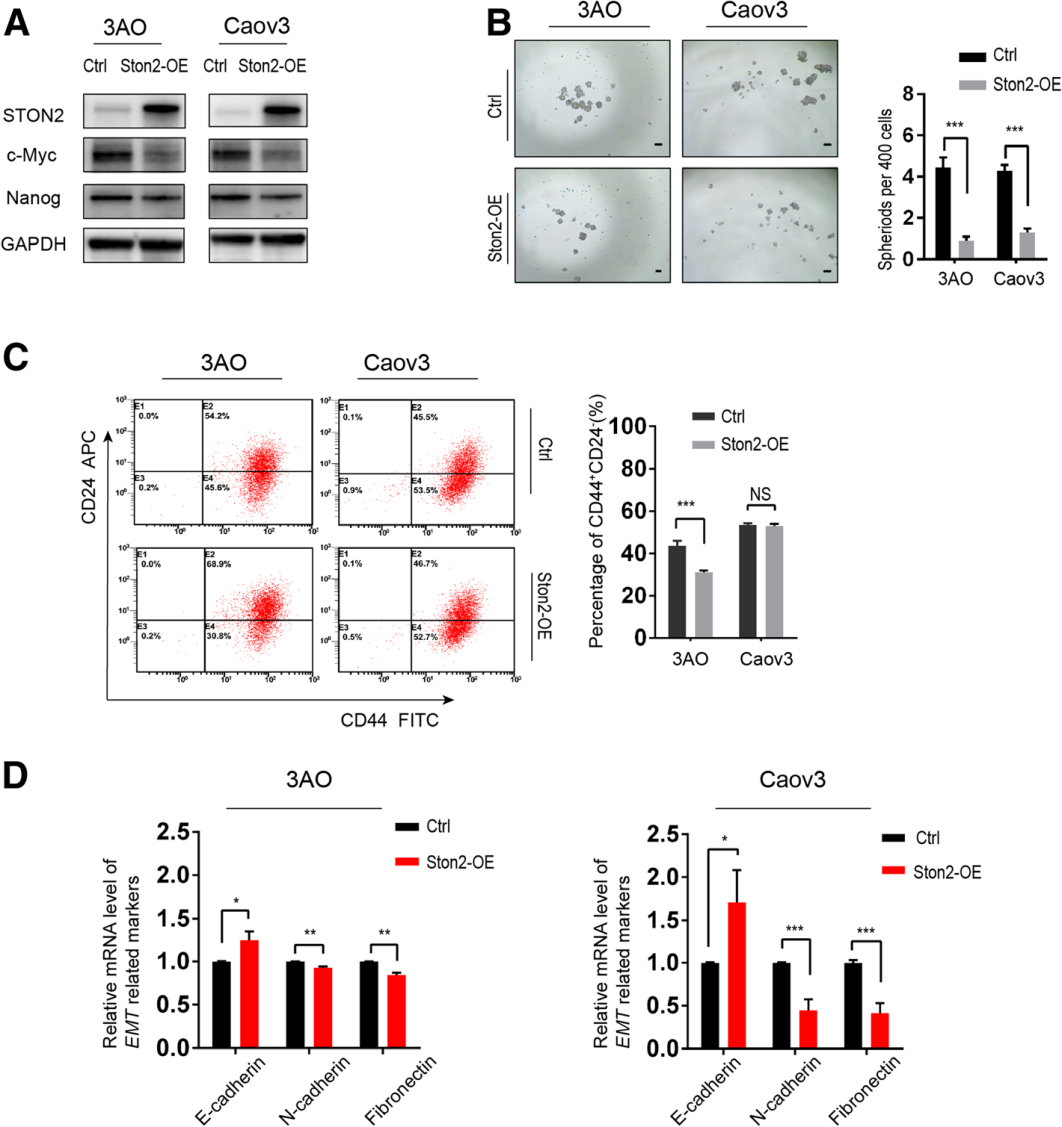
STON2 overexpression inhibits stem-like properties of ovarian cancer cells**. a** 3AO and Caov3 cells were transfected with STON2 overexpressing plasmid for 48 h and cultured in spheroid culture conditions for 7 days. Expression of STON2 and the CSC-related markers NANOG and c-MYC were determined by immunoblot analysis. **b** Quantitation and representative images of sphere-formation (sphere > 50 μm), Scale bars, 50 μm. **c** Effect of STON2 overexpression on the CD44^+^CD24^−^ phenotype was analyzed using FCM. **d** qPCR analysis of EMT-related markers (E-cadherin, N-cadherin, and fibronectin). Data represents the mean ± S.E. of three independent experiments. The level of significance is indicated by *P < 0.05, **P < 0.01, ***P < 0.001, NS indicates *P* > 0.05

Next, we considered whether *STON2* overexpression negatively affects OCSCs. 3AO and Caov3 cells were transfected with STON2 plasmids and cultured in SFM for 7 days. Cells transfected with the empty vectors acted as controls. Analysis of the expression of CSC-related proteins showed that their expression levels were reduced in cells showing *STON2* overexpression (Fig. 3a). *STON2* overexpression also significantly inhibited spheroid number (Figs. 3b) and the proportion of cells with the CD44^+^CD24^−^phenotype in the 3AO cell line, but not in the Caov3 cell line (Fig. 3c). It also altered the expression of EMT-related key factors (Fig. 3d), as compared to those of the control groups.

We then investigated the effect of *STON2* knockdown on ovarian tumorigenicity. We performed xenograft assays using different gradients of CSC subpopulations enriched from 3AO-shNC or shSTON2 cells. As shown in Fig. 2f, by 4 weeks after injection, 1 × 10^4^ shSTON2 cells had efficiently generated large tumors in all mice (*n* = 5), but equal numbers of shNC cells had produced only three tumors among five injected NOD/SCID mice. These data indicated that *STON2* knockdown enhanced the CSC subpopulation-induced tumorigenicity in vivo. In addition, the tumor diameters of mice injected with 1 × 10^5^ cells were measured weekly for 4 weeks after implantation. As shown in Fig. 2g, the volume of tumors in the shSTON2 groups was larger than those in the shNC groups.

### MUC1 acts as a downstream target for STON2

**Fig. 4:**
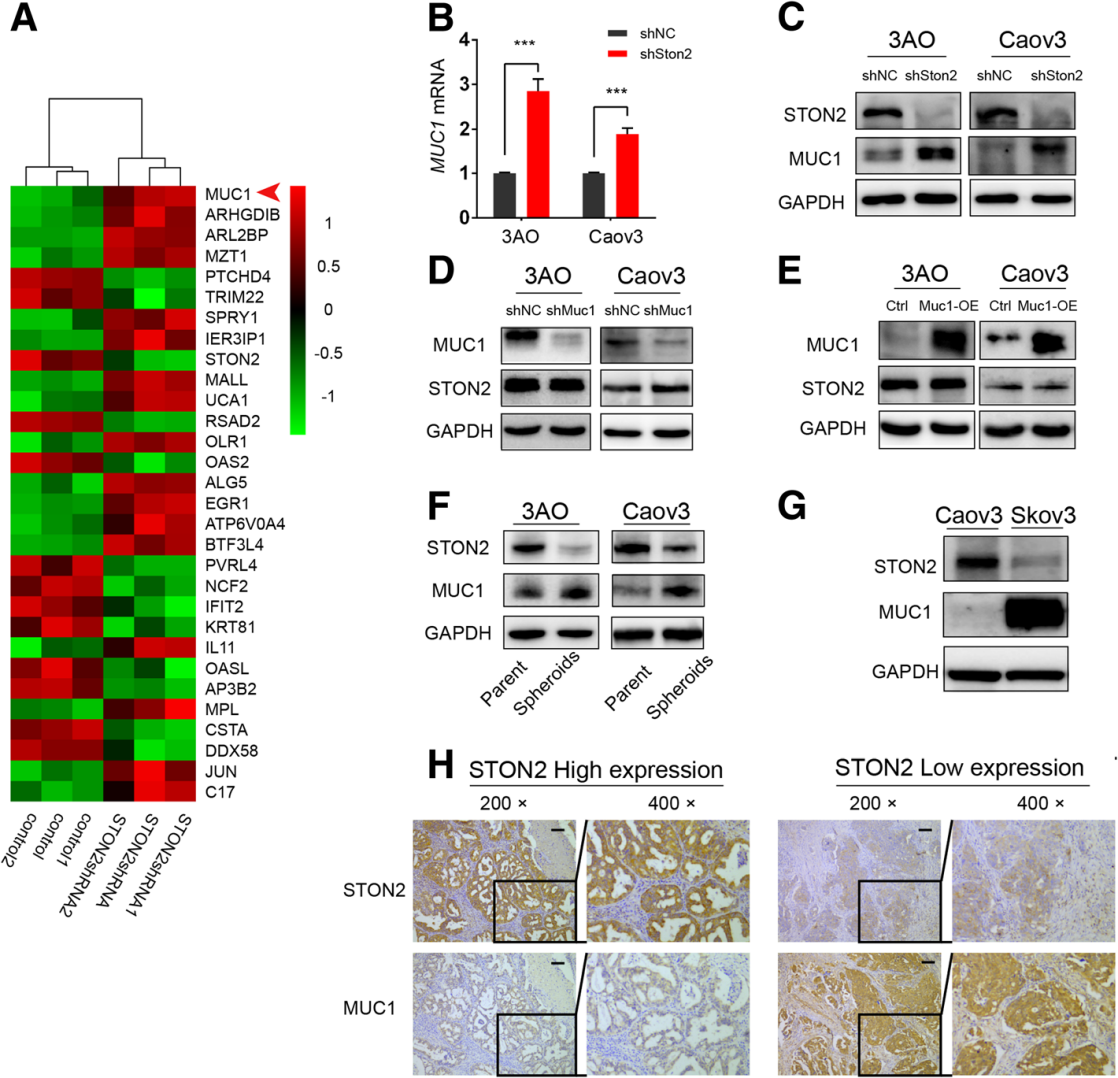
MUC1 acts as a downstream target for STON2**. a** RNA-seq data from the analysis between shNC and shSTON2 of 3AO cells derived stem cells. The cluster map generated by DESeq2 software analysis shows a series of differentially expressed genes (Padj < 0.05). Genes up-regulated in shSTON2 are shown in red, and down-regulated genes in green. The intensity of the color green or red corresponds to the degree of alteration, according to the color strip at the right of the figure. The arrow indicates MUC1. **b** qPCR analysis of MUC1 mRNA expression regulated by STON2 in 3AO and Caov3. **c** Immunoblot analysis of MUC1 protein level regulated by STON2 in 3AO and Caov3. **d** STON2 protein level after MUC1 knockdown in 3AO and Caov3. **e** STON2 protein level after MUC1 overexpression in 3AO and Caov3. **f** Immunoblot analysis of STON2 and MUC1 expression in parental cells and spheroids. **g** Immunoblot analysis of STON2 and MUC1 expression in Caov3 and Skov3 cells. **h** Specimen sections from the paraffin-embedded block from ovarian cancer patients were used for detection using anti-STON2 and anti-MUC1 antibodies, respectively. Paired STON2 and MUC1 staining is shown at 200× and 400× magnifications. Data represents the mean ± S.E. of three independent experiments. The level of significance is indicated by ***P < 0.001

As STON2 modulates stem-like properties in ovarian cancer cells, we attempted to determine the downstream targets of STON2. Total RNA was extracted from spheroid cells treated with shNC or shSTON2, and the differential mRNA expression was detected using RNA-seq (three independent samples in each group). DESeq2 software analysis showed that a series of genes was differentially expressed (< 0.05-Padj) (Fig. 4a). We focused on MUC1, which was shown to be particularly up-regulated (− 0.0000446-Padj) and has been demonstrated to be related to stemness in breast cancer [28] and pancreatic cancer [29].

We determined MUC1 expression in 3AO and Caov3 cells using qPCR and western blotting. Results showed that the expression levels of MUC1 mRNA and protein were elevated after *STON2* knockdown (Fig. 4b, c), whereas, STON2 expression remained unaltered upon *MUC1* knockdown or over-expression (Fig. 4d, e). This suggests that MUC1 acts downstream of STON2 in ovarian cancer cells.

We also analyzed the expression of MUC1 in parental as well as in cancer cell derived stem cells. Western blotting showed a higher expression of MUC1 associated with a lower expression of STON2 in spheroids, as compared to those in parental cells (Fig. 4f). We also employed a highly aggressive ovarian cancer cell line Skov3 [30] to examine MUC1 expression. As expected, a much higher expression of MUC1 was associated with a lower expression of STON2 in the Skov3 cells, as compared to those in Caov3 cells (Fig. 4g).

Further random examinations of specimen sections from the paraffin-embedded blocks of ovarian cancer patients confirmed that STON2 and MUC1 expression correlated negatively (Fig. 4h).

### DNMT1-MUC1 mediated epigenetic modification contributes to STON2 regulation in ovarian cancer cells

**Fig. 5:**
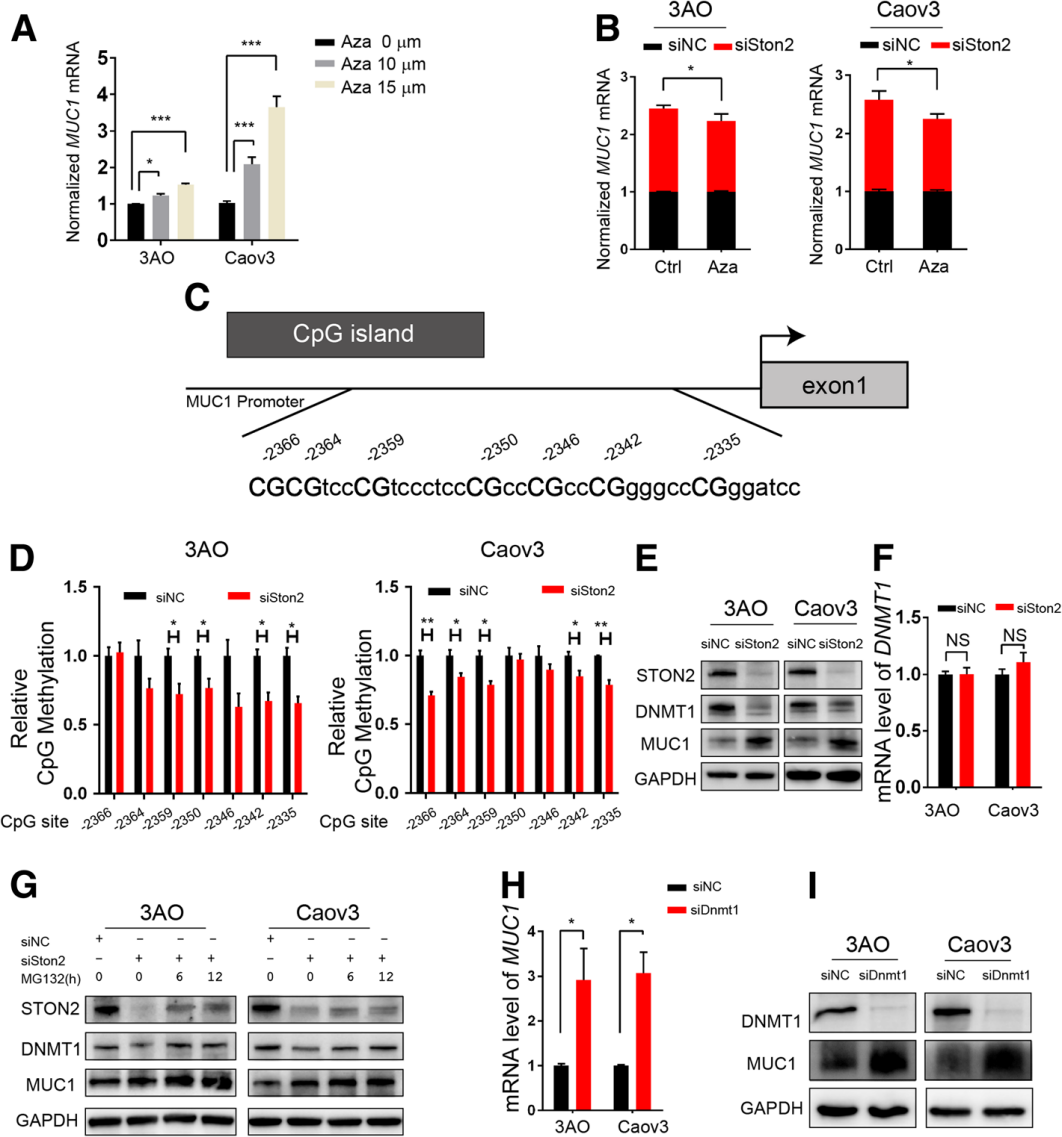
DNMT1-MUC1 mediated epigenetic modification contributes to STON2 regulation in ovarian cancer cells.** a** 3AO and Caov3 cells were treated with 0 μM, 10 μM, and 15 μM Aza (S1782, Selleck, USA) for 48 h, and *MUC1* expression was detected using qPCR analysis. **b** 3AO and Caov3 cells were transfected with a STON2-specific siRNA or siNC for 24 h, followed by treatment with Aza (15 μM for 48 h). *MUC1* expression was detected using qPCR analysis. **c** Schematic illustration of the *MUC1* promoter embedded in one CpG-rich region (− 2366 to − 2327 bp). **d** 3AO and Caov3 cells were transfected with a STON2-specific siRNA or control for 24 h, followed by pyrosequencing to assess the DNA methylation status of *MUC1* (three coupled independent samples). Each column represents the relative average DNA methylation level at one CpG site compared to the control group (see also raw pyrograms of representative experiments in Additional file 5: Figure S2). **e**-**g** 3AO and Caov3 cells were transfected with a STON2-specific siRNA for 72 h. DNMT1 and MUC1 expression were detected by immunoblot analysis (**e**). *DNMT1* expression was determined by qPCR analysis (**f**). DNMT1 and MUC1 expression was assayed by immunoblotting in the presence of MG132 (10 μM for 0 h, 6 h, 12 h) **g**. (h-i) 3AO and Caov3 cells were transfected with a DNMT1-specific siRNA for 72 h. *MUC1* expression was detected using qPCR analysis (**h**). MUC1 expression was detected using immunoblot analysis (**i**). Data represents the mean ± S.E. of three independent experiments. The level of significance is indicated by *P < 0.05, **P < 0.01, ***P < 0.001, NS indicates *P* > 0.05

Previous studies have shown that *MUC1* expression is regulated by DNA methylation [24, 31]. Hence, we treated 3AO and Caov3 cells with different concentrations of DNA methylation inhibitor azacitidine (Aza) for 48 h. An Aza dosage-dependent increase in *MUC1* expression was observed (Fig. 5a). We then transfected a STON2-specific siRNA or control siRNA in 3AO and Caov3 cells and incubated them for 24 h. This was followed by treatment with Aza or control for 48 h. Results showed that Aza was able to partially block *MUC1* up-regulation post-*STON2* knockdown (Fig. 5b), suggesting that *STON2* knockdown-induced *MUC1* up-regulation might occur via a reduction in *MUC1* promoter methylation. We further identified one CpG-rich region in the *MUC1* promoter (Fig. 5c) and designed primers to analyze the CpG-rich region using a PyroMark assay design. 3AO and Caov3 cells were treated with Aza for 24 h after transfection with the STON2-specific siRNA or control siRNA. The DNA methylation levels of the specific CpG nucleotides were then detected using pyrosequencing. Results showed that the extent of DNA methylation at multiple sites in the siSTON2 group was less than that of the control group (Fig. 5d and Additional file 5: Figure S2).

DNMT1, a key member of the DNA methyltransferase family [32], is known to maintain methylation patterns and suppress transcription [33]. Our results showed that *STON2* knockdown decreased DNMT1 but increased MUC1 expression (Fig. 5e). We also observed that *STON2* knockdown did not alter *DNMT1* mRNA levels but changed DNMT1 protein levels (Fig. 5e, f). Growing evidence suggests that the stability of DNMT1 is regulated by post-translational modifications such as phosphorylation, acetylation, methylation, and ubiquitination [34–36]. Using specific siRNAs, we confirmed that the *STON2* knockdown-induced reduction in DNMT1 could be partially rescued by the proteasome inhibitor MG132, and that a time-dependent increase in MUC1 expression was observed (Fig. 5g). In addition, both the mRNA expression and protein level of MUC1 were increased by *DNMT1* knockdown (Fig. 5h, i). Correspondingly, the *MUC1* promoter DNA methylation levels of specific CpG nucleotides at multiple sites in the siDNMT1 group were lower than those in the control group (Additional file 6: Figure S3). These results suggest that, for cancer stem cells, STON2 functions at the post-transcriptional level via the DNMT1/MUC1 pathway.

### MUC1 participates in the STON2-mediated modulation of stem-like properties in ovarian cancer cells

**Fig. 6:**
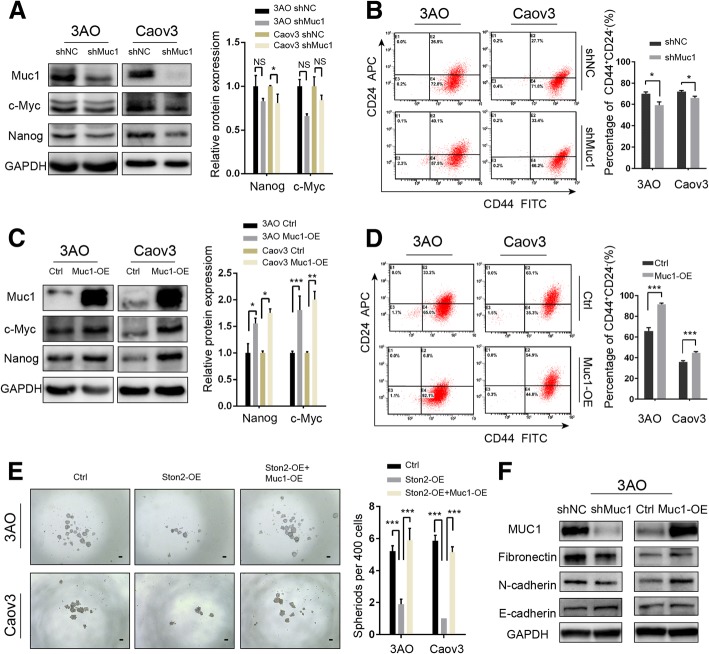
MUC1 participates in the STON2-mediated modulation of stem-like properties in ovarian cancer cells. **a**, **b** 3AO and Caov3 cells were transfected with a MUC1-specific shRNA for 48 h and cultured in spheroid culture conditions for 7 days. Representative images and the relative expression levels of CSC-related markers NANOG and c-MYC were detected by immunoblot analysis (**a**), and the CD44^+^CD24^−^ population was detected by FCM (**b**). **c**, **d** 3AO and Caov3 cells were transfected with *MUC1* overexpressing plasmid for 48 h, and cultured in spheroid culture conditions for 7 days. Representative images and the relative expression levels of CSC-related markers NANOG and c-MYC were detected by immunoblot analysis (**c**), and the CD44^+^CD24^−^ population was detected using FCM (**d**). **e** 3AO and Caov3 cells were transfected with a control vector, *STON2* overexpression plasmid, or *STON2* overexpression plasmid plus *MUC1* overexpression plasmid for 48 h and cultured in spheroid culture conditions for 7 days. Sphere-formation (sphere> 50 μm) was assessed. Scale bars, 50 μm. **f** 3AO cells were transfected with a MUC1-specific shRNA, or *MUC1* overexpressing plasmid for 48 h, and cultured in spheroid culture conditions for 7 days. EMT-related markers (E-cadherin, N-cadherin, and fibronectin) were detected using immunoblot analysis. Data represents the mean ± S.E. of three independent experiments. The level of significance is indicated by *P < 0.05, ***P < 0.001

Finally, we constructed shRNA to suppress *MUC1* in 3AO and Caov3 cells cultured in SFM. We analyzed the relative expression levels of CSC-related proteins, NANOG and c-MYC. The results showed that *MUC1* knockdown significantly reduced the expression level of NANOG in Caov3 (*p* < 0.05), but not in 3AO (Fig. 6a). The reduction of c-MYC expression was not significant in 3AO or Caov3 cells (Fig. 6a). *MUC1* knockdown also significantly reduced CD44^+^CD24^−^phenotypes (Fig. 6b). Conversely, *MUC1* overexpression significantly up-regulated both NANOG (*p* < 0.05) and c-MYC (*p* < 0.01) (Fig. 6c) and increased the proportion of cells with the CD44^+^CD24^−^ phenotype (Fig. 6d). Furthermore, we observed that the *STON2* overexpression-induced reduction of sphere-forming ability was partially reversed by *MUC1* overexpression (Fig. 6e).

Using *MUC1* knockdown or overexpression in the 3AO cell line, we detected the expression levels of EMT-related key factors by western blotting. As expected, the results showed that the expression of E-cadherin increased, while N-cadherin and fibronectin decreased in shMUC1 cells, as compared to those of the shNC cells (Fig. 6f). *MUC1* overexpression up-regulated the expression of N-cadherin and fibronectin, but did not significantly change E-cadherin, as compared to those of the control groups (Fig. 6f). However, we failed to co-express the shSTON2 and shMUC1 in 3AO cells. Based on previous results that the EMT-related protein expression was regulated by shSTON2 (Fig. 2d), it is conceivable that MUC1 participates in STON2 induced changes in EMT-related markers.

### The clinical significance of MUC1 and STON2 expression in ovarian cancer patients

**Fig. 7:**
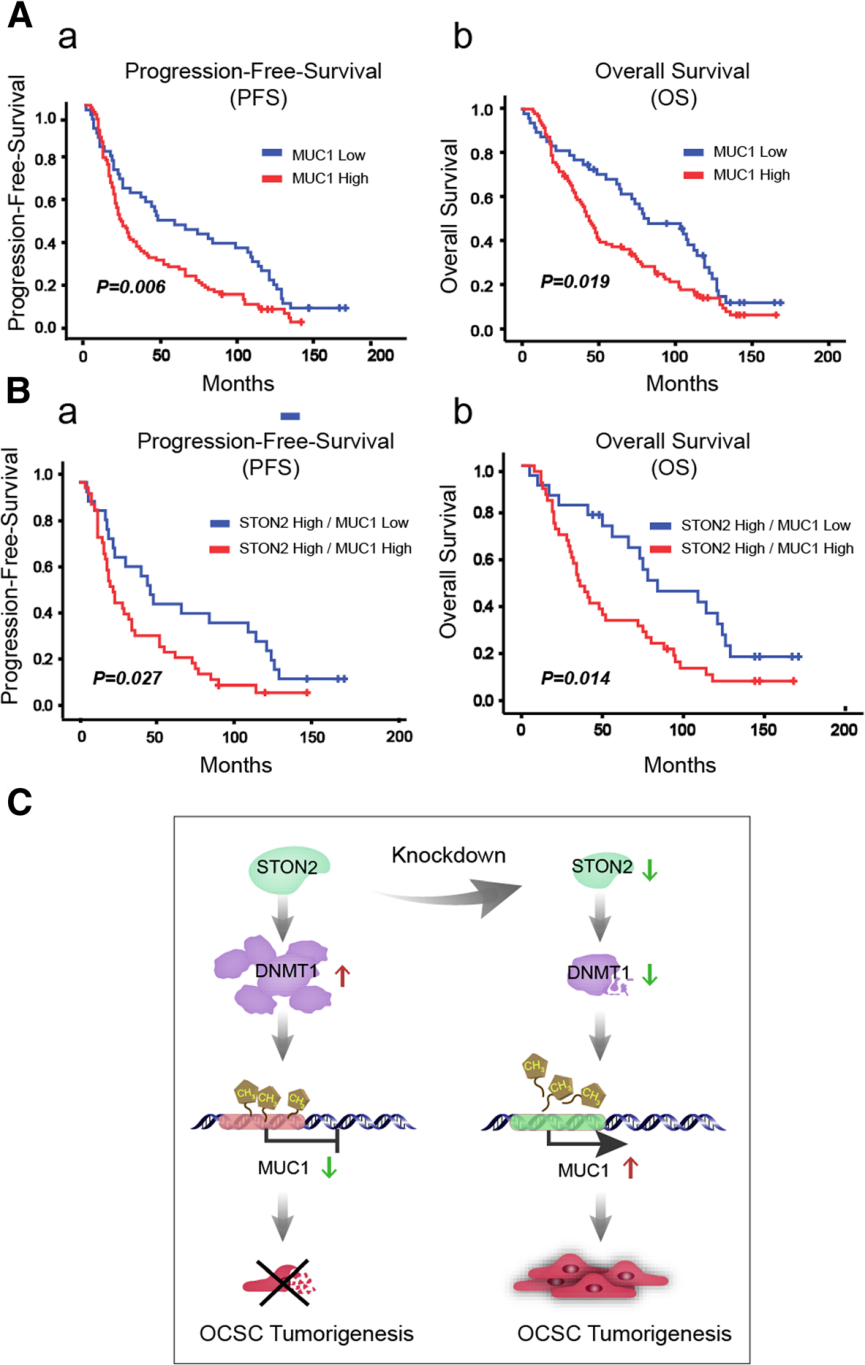
The clinical significance of MUC1 and STON2 expression in ovarian cancer patients. **a**, **b** Cumulative survival probabilities (a, PFS and b, OS) were calculated using the Kaplan–Meier method (*n* = 145) based on STON2 and MUC1 expression. Survival rates were compared using log-rank test. Patients expressing low or high levels of MUC1 (**a**). Patients expressing high levels of STON2 combined with low MUC1 expression or high MUC1 expression (**b**). **c** A schematic model of the role of STON2 in regulating CSCs traits

We firstly investigated the correlation between STON2 expression and clinical parameters based on immunohistological examination of tissue samples from patients. Kaplan–Meier survival analysis failed to reveal any correlation of STON2 expression with progression-free survival (PFS) or overall survival (OS) in our patient cohort (Additional file 7: Figure S5A). However, significantly lower STON2 immunoreactivity was detected in poorly differentiated ovarian cancer (*P* = 0.000, Additional file 8: Table S4). We also analyzed the correlation of MUC1 expression with clinicopathological characteristics, and prognosis of ovarian cancer. Results showed that significantly higher expression of MUC1 was associated with FIGO stage (*P* = 0.004), tumor grade (*P* = 0.01), serum CA125 (*P* = 0.005), and PFS (*P* = 0.006), OS (*P* = 0.019) (Additional file 9: Table S5 and Fig. 7a).

IHC analysis of the co-expression relationships between STON2 and MUC1 revealed that the prognostic value of STON2 expression was highly dependent on MUC1 expression, in which the high expression of STON2 twinned with a low expression of MUC1 was associated with a favorable prognosis (Fig. 7b). A trend was also revealed in that patients expressing high STON2 and low MUC1 were associated with a best survival of all the remaining patients (PFS, *P* = 0.095, and OS, *P* = 0.06). However, with a low expression of STON2, there was no prognostic value of MUC1 expression (Additional file 7: Figure S5B). Taken together, these observations provide evidence that STON2 acts as a negative modulator via the MUC1-mediated pathway in ovarian cancer.

## Discussion

We have shown that the OCSC-enriched population displays a dramatically decreased STON2 expression. STON2 knockdown promotes stem-like properties in ovarian cancer cells and its overexpression suppresses MUC1-induced sphere formation in OCSCs, in which DNMT1-mediated methylation in the promoter region of *MUC1* has been confirmed to be modulated by STON2. Our findings suggest that STON2 depression up-regulates MUC1 expression, along with inhibition of DNMT1, in a process that contributes to the maintenance of stem-like properties in ovarian cancer cells (Fig. 7c).

In our study, *STON2* knockdown increased both MUC1 mRNA and protein levels. This suggests that the regulation of MUC1 by STON2 occurs at the transcriptional level. *MUC1* expression has been reported to be associated with DNA methylation [23, 24, 37]. MassARRAY assays have been used to detect high methylation levels in the *MUC1* promoter region in MDA-MB-453, a MUC1-negative cancer cell line. *MUC1* mRNA expression in MUC1-negative cells has been shown to be restored by treatment with a DNA methylation inhibitor. MCF-7, a MUC1-positive cell line, has been associated with a low methylation level in the *MUC1* promoter region [24].

DNA methylation is an important genome modification that occurs at the cytosine residues within CpG dinucleotides. There are three enzymatically active mammalian methyltransferases: DNMT1, DNMT3A, and DNMT3B [38]. DNMT1 is responsible for maintaining methylation patterns in CpG dinucleotide-rich regions as well as for transcriptional repression [39], whereas DNMT3A and DNMT3B are involved in de novo methylation [40]. In the present study, we observed that only DNMT1, but not DNMT3A or DNMT3B, responded to *STON2* knockdown, (Additional file 10: Figure S4). Subsequently, *STON2* knockdown suppressed DNMT1 and increased MUC1 expression. Pyrosequencing data also revealed that the methylation level was reduced in the *MUC1* promoter region in *STON2* or *DNMT1* knocked down ovarian cancer cells. Therefore, we are the first to demonstrate that STON2 is involved in the DNMT1-mediated epigenetic modification of MUC1 promoter methylation in OCSCs.

Compared to other tumor types, for which there is a more generalized consensus on the CSC-related markers (such as for CD44^+^CD24^−^ in breast carcinomas, CD133^+^ in glioblastomas, and LGR5^+^ in colon carcinomas, etc.), it is striking to see how heterogeneous the sets of putative OCSC markers are [41], particularly, CD44, CD133, CD117, ALDH1 [42], and the combinations CD44^+^/CD24^−^, CD44^+^/CD117^+^ [43]. Isolated OCSCs are often characterized by the expression of stemness-associated genes such as NANOG [44] and c-MYC [11], both of which play key roles in maintaining the pluripotency of embryonic stem cells [45, 46]. Similarly, CSCs isolated from ascites derived from ovarian cancer patients showed elevated NANOG expressions [47]. NANOG has been widely used as a stem cell marker [48], is associated with stemness maintenance [12] and EMT acceleration [44], and is taken as an indicator of a poorer prognosis in ovarian cancers [49]. In addition, c-MYC expression has been most often proposed as a general feature of the CSCs of various cancers including breast [50], prostate [51], esophagus [52] and tongue [53], and the expression of c-MYC together with NANOG has been previously noted as a feature in a population of ovarian cancer cells [27]. In our previous study [9], we successfully enriched and characterized OCSCs with CD44^+^CD24^−^. Interestingly, here we showed that NANOG and c-MYC expression, as stemness-associated factors, were detected in these OCSCs and that their cancer stem-like properties were negatively regulated by *STON2* expression.

In the present study, we found that analysis of STON2 in isolation did not have any prognostic value in ovarian cancer. This provides a contrast to a previously published finding that STON2 overexpression is correlated with unfavorable prognosis in 89 ovarian cancer patients [54]. The reason for such an inconsistency is unclear, but our paper certainly represents a fairly comprehensively exploration of the prognostic value of STON2 expression and MUC1 expression in ovarian cancer. Conversely, we found that the prognostic value of high STON2 expression was highly dependent on MUC1 expression, with a trend that if a high expression of STON2 occurred together with a low expression of MUC1, then this was positively associated with a favorable prognosis. A clinical study based on larger patient samples is required for further confirmation.

## Conclusions

In summary, we observed for the first time that STON2 acts as a negative modulator in ovarian cancer cells via DNMT1/MUC1-mediated epigenetic mechanisms. STON2, is therefore involved in OCSC biology and may represent a therapeutic target for innovative treatments aimed at ovarian cancer eradication.

## Additional files

Additional file 1: **Table S1.** Primer sequences used in this study (DOCX 18 kb)

Additional file 2: **Table S2.** Target sequences in this study. (DOCX 18 kb)

Additional file 3: **Figure S1.** Intensity of STON2 and MUC1 immunohistochemical staining in ovarian cancer tissues. Representative images of STON2 and MUC1 staining in ovarian cancer tissues are shown. Scale bars represent approximately 50 μm. Staining intensity is graded as follows: 1+, weak; 2+, moderate; 3+, strong; 4+, very strong. (PNG 4594 kb)

Additional file 4: **Table S3.** Peptide information from the LC-MS/MS data after analyzing parental cells and spheroids of 3AO cells (FDR < 0.05). Av. Ratio (Lin): the ratio of spheroids to parental cells. (XLS 154 kb)

Additional file 5: **Figure S2.** (Related to pyrosequencing data shown in Fig. 5d) Representative pyrogramms of *MUC1* promoter in 3AO and Caov3 cells (siNC or siSTON2). (TIF 760 kb)

Additional file 6: **Figure S3.** 3AO and Caov3 cells were transfected with a DNMT1-specific siRNA or controls for 24 h, and then subjected to pyrosequencing to assess the DNA methylation status of *MUC1* (three coupled independent samples) (A-B). Each column represents the relative average DNA methylation level at one CpG site compared to the control group (A). Raw pyrograms of representative experiments (B). (TIF 1569 kb)

Additional file 7: **Figure S5.** (A-B) Cumulative survival probabilities (a, PFS and b, OS) were calculated using the Kaplan–Meier method (*n* = 145) based on STON2 and MUC1 expression. Survival rates were compared using a log-rank test. Patients expressing low or high levels of STON2 (A). Patients expressing low levels of STON2 combined with low MUC1 expression or high MUC1 expression (B). (TIF 912 kb)

Additional file 8: **Table S4.** Association of STON2 expression with clinicopathological characteristics in patients with ovarian cancer (*n* = 165). (XLSX 11 kb)

Additional file 9: **Table S5.** Association of MUC1 expression with clinicopathological characteristics in patients with ovarian cancer (*n* = 165). (XLSX 11 kb)

Additional file 10: **Figure S4.** 3AO and Caov3 cells were transfected with a STON2-specific siRNA for 72 h, and the expression of DNMT1 (A), DNMT3A (B), or DNMT3B (C) was assayed using immunoblotting analysis. (TIF 808 kb)

## Abbreviations

ARRIVE: Animal research reporting in vivo experiments
ATCC: American type culture collection
Aza: azacitidine
CSCs: Cancer stem cells
CSLCs: Cancer stem-like cells
DMEM: Dulbecco’s modified eagle’s medium
DNMT1: Dna methyltransferase 1
ECL: Enhanced chemiluminescence
EMT: Epithelial mesenchymal transition
FBS: Fetal bovine serum
FCM: Flow cytometry analysis
FDR: False discovery rate
FIGO: Federation international of gynecology and obstetrics
IHC: Immunohistochemistry
IPI: International protein index
KEGG: Kyoto encyclopedia of genes and genomes
LC: Liquid chromatography
MS: Mass spectrometry
MUC1: Mucin1
OCSCs: Ovarian cancer stem cells
OS: Overall survival
PFS: Progression-free survival
PVDF: Polyvinylidene fluoride
qPCR: Quantitative real-time polymerase chain reaction
RIPA: Radioimmunoprecipitation assay
RNA-Seq: RNA-sequencing
RPMI: Roswell park memorial institute
SFM: Serum-free medium
shRNA: Short-hairpin RNA
siRNAs: Small-interfering RNAs
STON2: Stonin2
TBST: Tris buffered saline Tween 20
TCGA: The cancer genome atlas
