# Supplementary material for: STON2 negatively modulates stem-like properties in ovarian cancer cells via DNMT1/MUC1 pathway
Source: J Exp Clin Cancer Res. 2018 Dec 5;37:305. doi: 10.1186/s13046-018-0977-y (PMC6282299; doi:10.1186/s13046-018-0977-y)
Supplement: Supplementary file 1 — Table S1. Primer sequences used in this study (DOCX 18 kb) [file 13046_2018_977_MOESM1_ESM.docx]

**Table S1: Primer sequences used in this study**

| Primers for RT-PCR | |
| --- | --- |
| *GAPDH* | Forward : 5’- GGAGCGAGATCCCTCCAAAAT-3’ |
|  | Reverse: 5’- GGCTGTTGTCATACTTCTCATGG-3’ |
| *STON2* | Forward : 5’- ACCTCTGCTCGTTTTCCCAG-3’ |
|  | Reverse: 5’- GGACGGTCCCTCTTCTTAAATG-3’ |
| *MUC1* | Forward : 5’- TGCCGCCGAAAGAACTACG-3’ |
|  | Reverse: 5’- TGGGGTACTCGCTCATAGGAT-3’ |
| *DNMT1* | Forward : 5’- CCTAGCCCCAGGATTACAAGG-3’ |
|  | Reverse: 5’- ACTCATCCGATTTGGCTCTTTC-3’ |
| Primers for pyrosequencing | |
| *MUC1* | Forward: 5’-GTGTAATATTGGAGGGGGAAAAAATAAT-3’ |
|  | Reverse：5’-CCAAATCTCTCTAACCTCCAAAATA-3’ |
|  | Sequence：GGGGGAAAAAATAATATTGTAT |
| Primers for plasmid sequencing | |
| *pcDNA3.1* | Forward : CTAGAGAACCCACTGCTTAC |
|  | Reverse: TAGAAGGCACAGTCGAGG |
