# Supplementary material for: STON2 negatively modulates stem-like properties in ovarian cancer cells via DNMT1/MUC1 pathway
Source: J Exp Clin Cancer Res. 2018 Dec 5;37:305. doi: 10.1186/s13046-018-0977-y (PMC6282299; doi:10.1186/s13046-018-0977-y)
Supplement: Supplementary file 2 — Table S2. Target sequences in this study. (DOCX 18 kb) [file 13046_2018_977_MOESM2_ESM.docx]

**Table S2: Target sequences in this study**

| Lentivirus target sequence | |
| --- | --- |
| *STON2* | GGTTTGAGCTAATGCGGTT |
| *MUC1* | CACAGUUCAAUCAGUAUAATT |
| Sequences of siRNA | |
| *STON2* | Forward : 5’- GGUUUGAGCUAAUGCGGUUTT-3’ |
|  | Reverse: 5’- AACCGCAUUAGCUCAAACCTT-3’ |
| *DNMT1* | Forward : 5’- GUCCCAAUAUGGCCAUGAATT-3’ |
|  | Reverse: 5’- UUCAUGGCCAUAUUGGGACTT-3’ |
