# Supplementary figures and images for: STON2 negatively modulates stem-like properties in ovarian cancer cells via DNMT1/MUC1 pathway
Source: J Exp Clin Cancer Res. 2018 Dec 5;37:305. doi: 10.1186/s13046-018-0977-y (PMC6282299; doi:10.1186/s13046-018-0977-y)

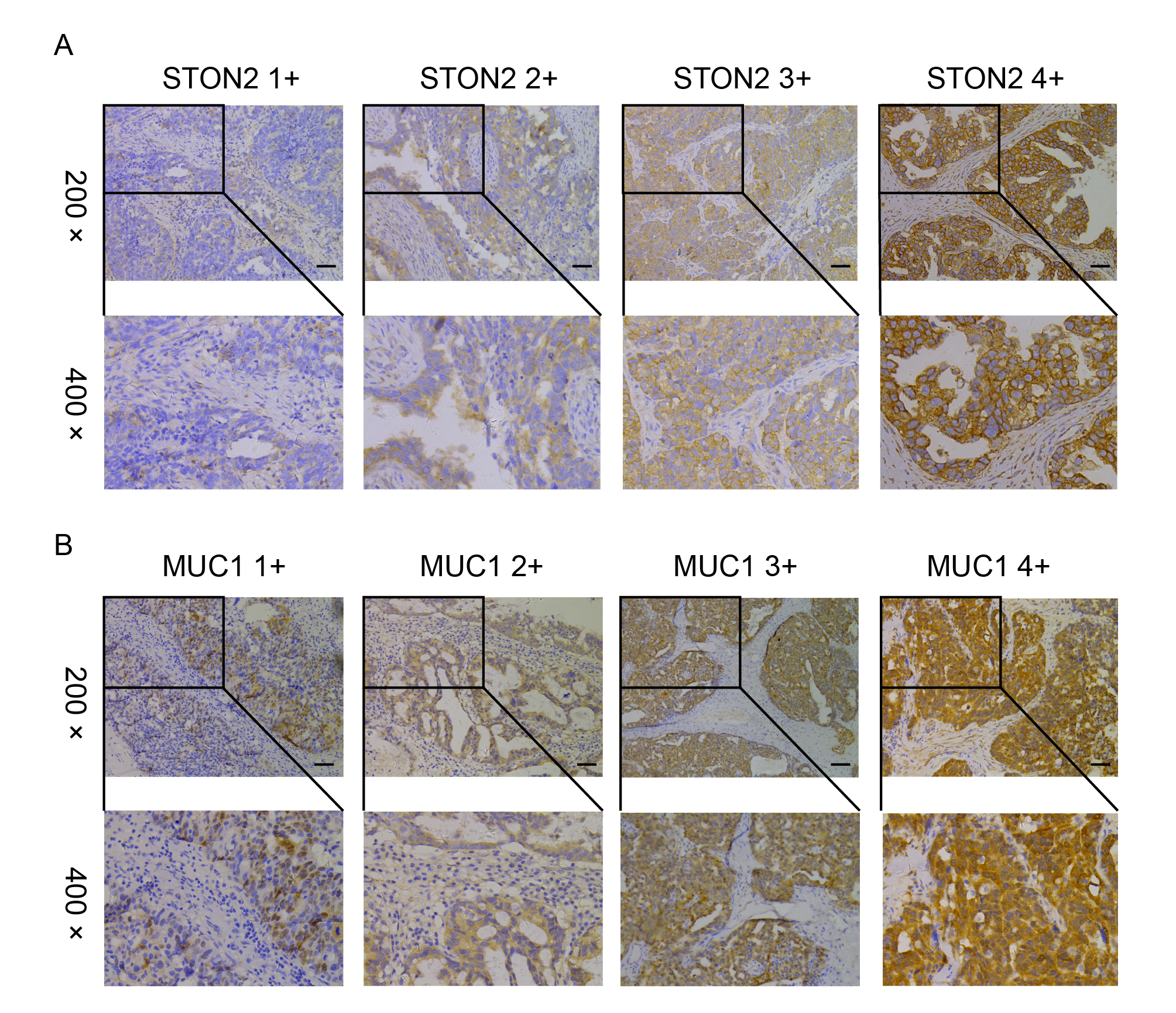

Supplement: Supplementary file 3 — Figure S1. Intensity of STON2 and MUC1 immunohistochemical staining in ovarian cancer tissues. Representative images of STON2 and MUC1 staining in ovarian cancer tissues are shown. Scale bars represent approximately 50 μm. Staining intensity is graded as follows: 1+, weak; 2+, moderate; 3+, strong; 4+, very strong. (PNG 4594 kb) [file 13046_2018_977_MOESM3_ESM.png]

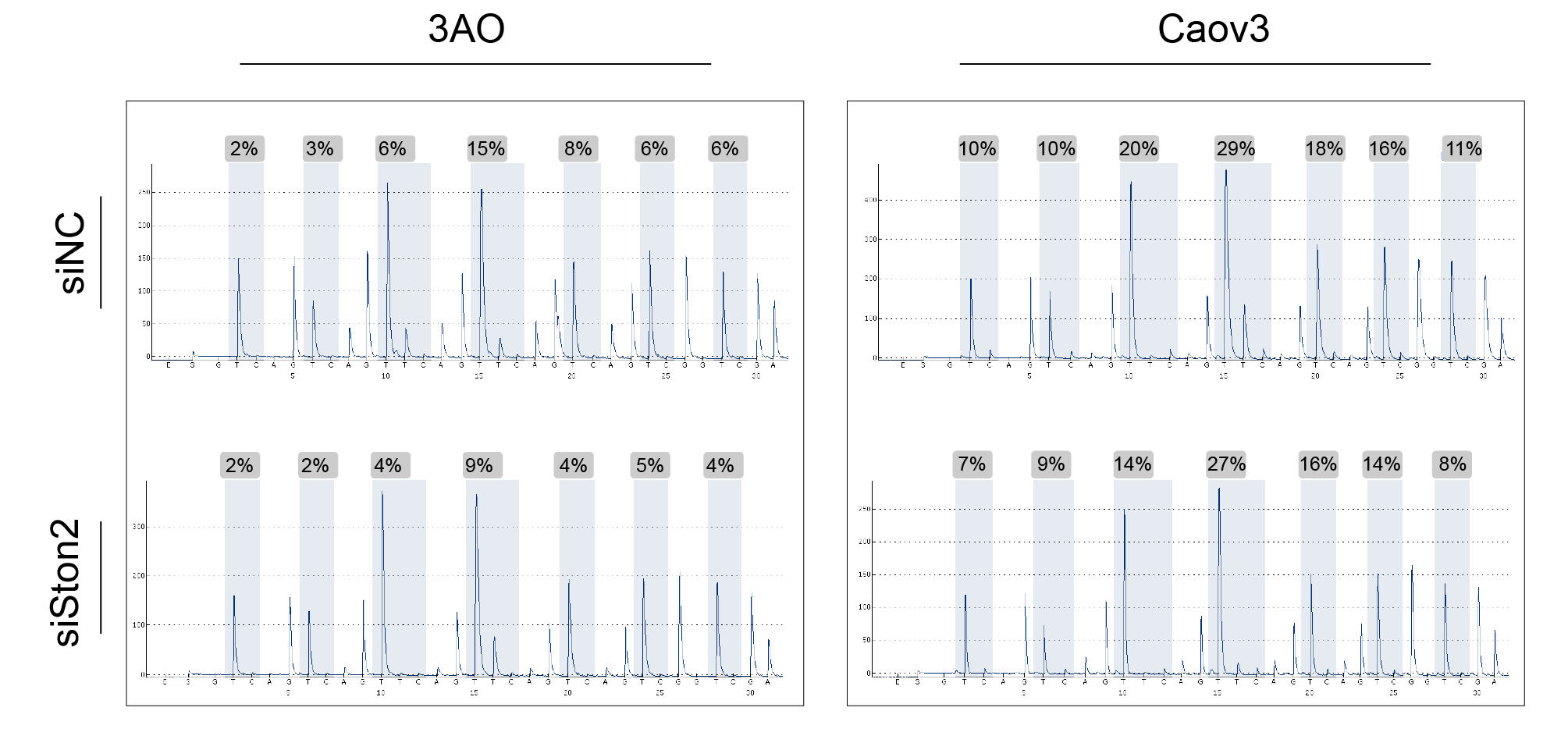

Supplement: Supplementary file 5 — Figure S2. (Related to pyrosequencing data shown in Fig. 5d) Representative pyrogramms of MUC1 promoter in 3AO and Caov3 cells (siNC or siSTON2). (TIF 760 kb) [file 13046_2018_977_MOESM5_ESM.tif]

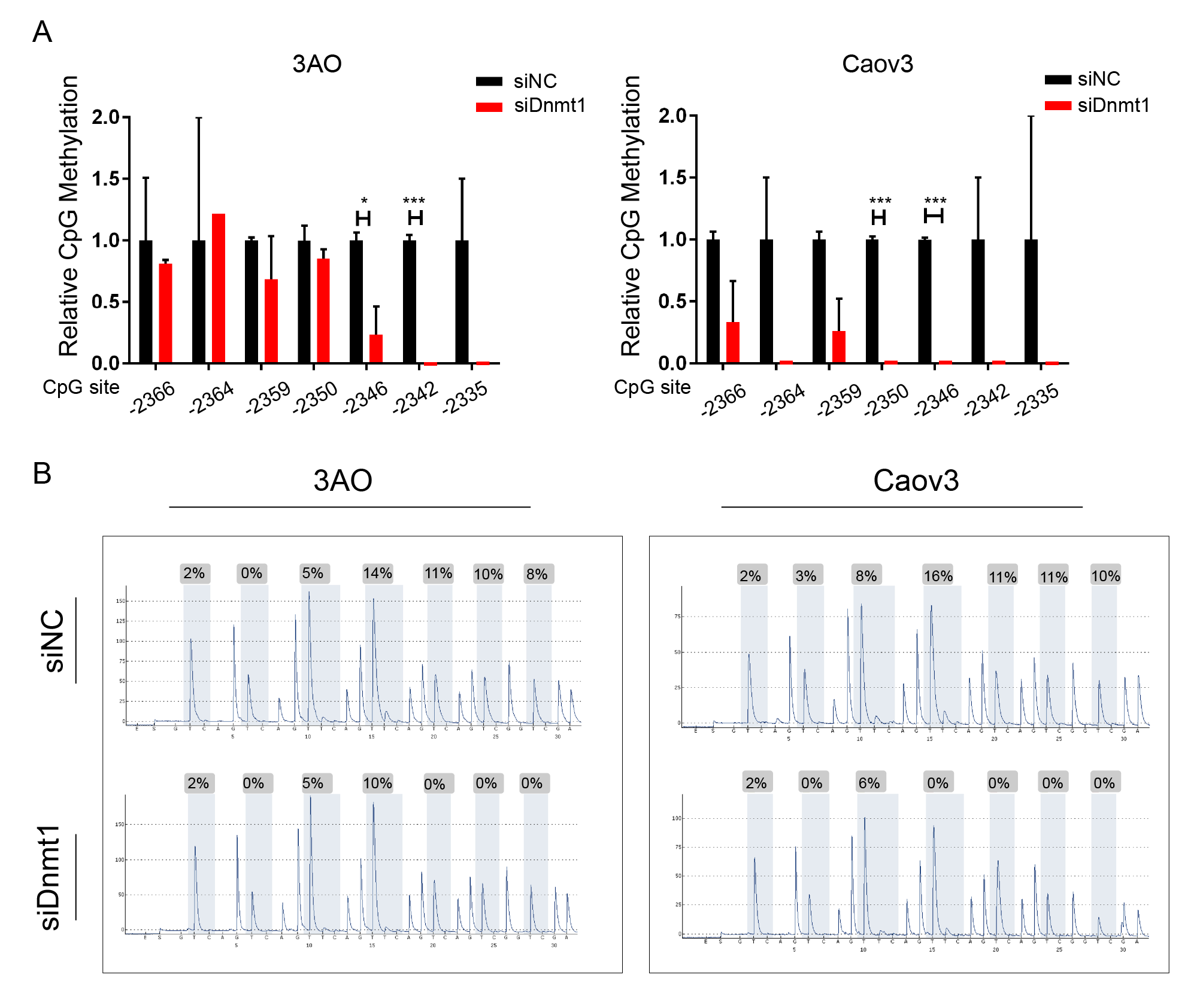

Supplement: Supplementary file 6 — Figure S3. 3AO and Caov3 cells were transfected with a DNMT1-specific siRNA or controls for 24 h, and then subjected to pyrosequencing to assess the DNA methylation status of MUC1 (three coupled independent samples) (A-B). Each column represents the relative average DNA methylation level at one CpG site compared to the control group (A). Raw pyrograms of representative experiments (B). (TIF 1569 kb) [file 13046_2018_977_MOESM6_ESM.tif]

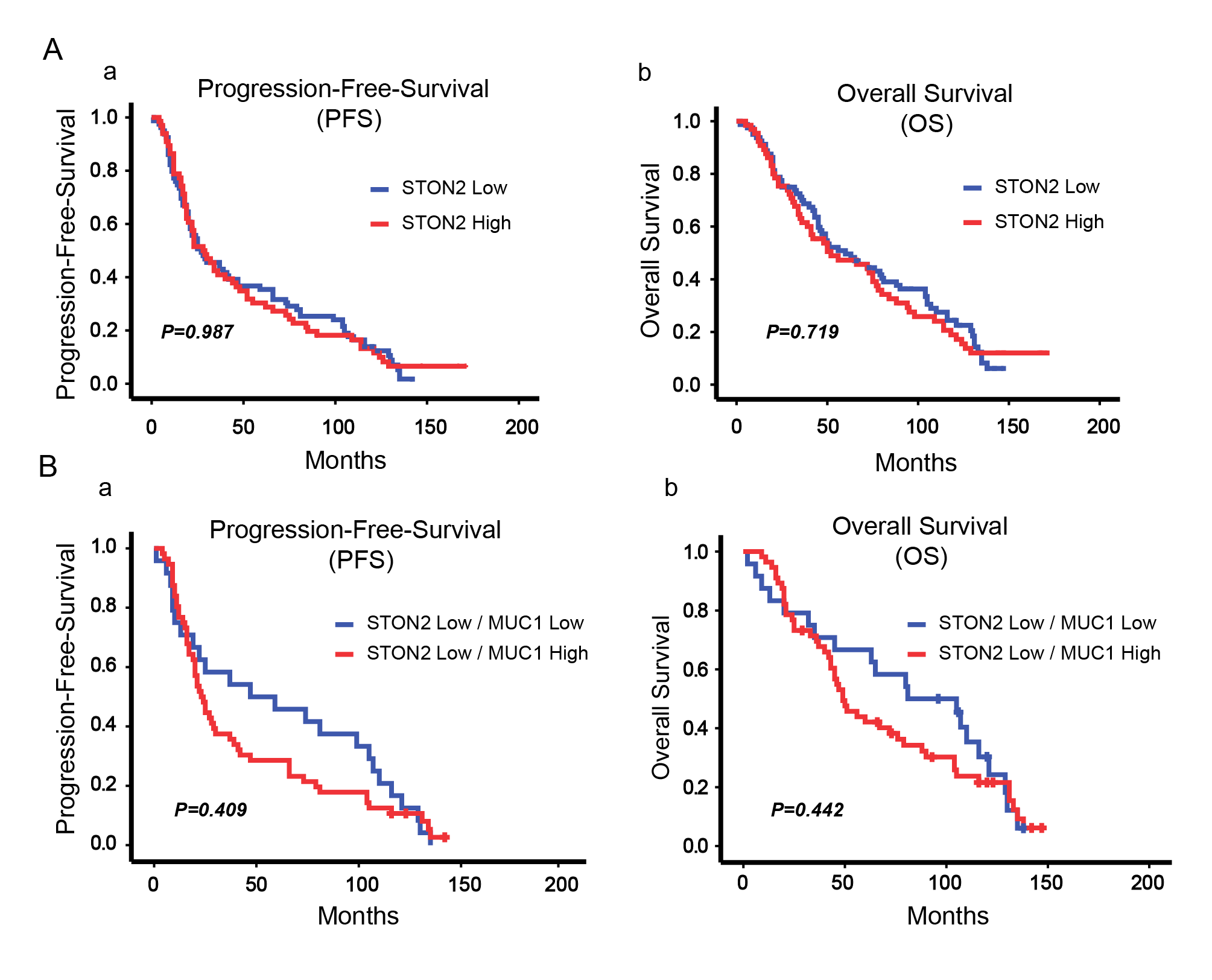

Supplement: Supplementary file 7 — Figure S5. (A-B) Cumulative survival probabilities (a, PFS and b, OS) were calculated using the Kaplan–Meier method (n = 145) based on STON2 and MUC1 expression. Survival rates were compared using a log-rank test. Patients expressing low or high levels of STON2 (A). Patients expressing low levels of STON2 combined with low MUC1 expression or high MUC1 expression (B). (TIF 912 kb) [file 13046_2018_977_MOESM7_ESM.tif]

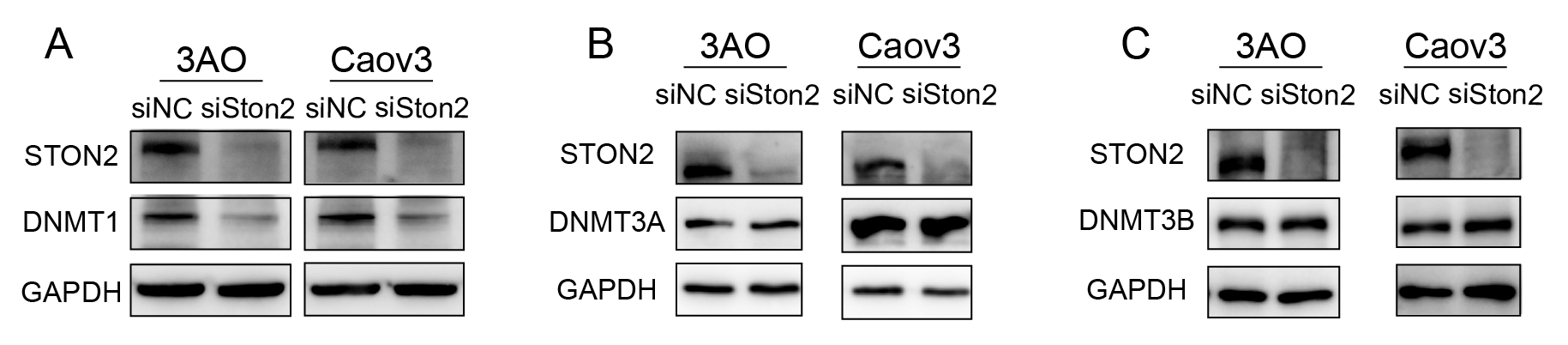

Supplement: Supplementary file 10 — Figure S4. 3AO and Caov3 cells were transfected with a STON2-specific siRNA for 72 h, and the expression of DNMT1 (A), DNMT3A (B), or DNMT3B (C) was assayed using immunoblotting analysis. (TIF 808 kb) [file 13046_2018_977_MOESM10_ESM.tif]
